# Spatial Charge Separation as the Origin of Anomalous Stark Effect in Fluorous 2D Hybrid Perovskites

**DOI:** 10.1002/adfm.202000228

**Published:** 2020-05-28

**Authors:** Valentin I. E. Queloz, Marine E. F. Bouduban, Ines García‐Benito, Alexander Fedorovskiy, Simonetta Orlandi, Marco Cavazzini, Gianluca Pozzi, Harsh Trivedi, Doru C. Lupascu, David Beljonne, Jaques‐E Moser, Mohammad Khaja Nazeeruddin, Claudio Quarti, Giulia Grancini

**Affiliations:** ^1^ Group for Molecular Engineering of Functional Materials Institute of Chemical Sciences and Engineering Ecole Polytéchnique Fédérale de Lausanne Sion CH‐1951 Switzerland; ^2^ Photochemical Dynamics Group Institute of Chemical Sciences and Engineering and Lausanne Centre for Ultrafast Science (LACUS) École Polytéchnique Fédérale de Lausanne Lausanne CH‐1015 Switzerland; ^3^ Consiglio Nazionale delle Ricerche Istituto di Scienze e Tecnologie Chimiche “Giulio Natta” (CNR‐SCITEC) Via Golgi 19 Milano I‐20133 Italy; ^4^ Institute for Materials Science and Center for Nanointegration Duisburg‐Essen (CENIDE) University of Duisburg‐Essen Essen 45141 Germany; ^5^ Laboratory for Chemistry of Novel Materials University of Mons Place du Parc 20 Mons B‐7000 Belgium; ^6^ ENSCR, INSA Rennes, CNRS Institut des Sciences Chimiques de Rennes (ISCR) University of Rennes UMR 6226 Rennes F‐35000 France; ^7^ Department of Chemistry and INSTM University of Pavia Via Taramelli 14 Pavia 27100 Italy

**Keywords:** 2D perovskite, electroabsorption, Stark effect, transient absorption

## Abstract

2D hybrid perovskites (2DP) are versatile materials, whose electronic and optical properties can be tuned through the nature of the organic cations (even when those are seemingly electronically inert). Here, it is demonstrated that fluorination of the organic ligands yields glassy 2DP materials featuring long‐lived correlated electron–hole pairs. Such states have a marked charge‐transfer character, as revealed by the persistent Stark effect in the form of a second derivative in electroabsorption. Modeling shows that electrostatic effects associated with fluorination, combined with the steric hindrance due to the bulky side groups, drive the formation of spatially dislocated charge pairs with reduced recombination rates. This work enriches and broadens the current knowledge of the photophysics of 2DP, which will hopefully guide synthesis efforts toward novel materials with improved functionalities.

## Graphical abstract

An anomalous, persistent, Stark effect from electroabsorption and nanosecond transient absorption measurement related to the presence of long‐lived screened electron–hole pairs in 2D perovskite is presented. A peculiarity in these materials, contrasting with large exciton binding energies, is presented, revealing a complex photophysical response and highlighting the role of cation's chemistry on the material's optical response.

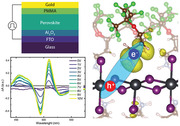

## 1 Introduction

Hybrid organic–inorganic perovskites (HP) are currently being scrutinized in view of their enormous potential for optoelectronic applications.^[^ ^1^, ^2^ ^]^ A peculiarity of this class of materials relies on their extreme structural versatility. In particular, by varying the organic component, the HP structure can be molded from a 3D arrangement to a 2D layered organization system, with consequently modified optoelectronic attributes and (photo)physics.^[^ ^3^, ^4^ ^]^ 2D perovskites (2DP) where single [PbI_4_]^2−^ inorganic layers are intercalated by large organic cations (R), in the form of R_2_PbI_4_, are nowadays captivating a growing interest. As quantum wells (QWs), they sustain stable excitons with relatively high Coulomb energy (Eb = 400–500 meV), due to quantum and dielectric confinement induced by the dielectric mismatch between the organic and inorganic moieties.^[^ ^5^, ^6^, ^7^, ^8^ ^]^

As a result of the strengthened exciton confinement, bright photoluminescence (PL), scintillation activity, and strong optical nonlinearities have been observed,^[^ ^9^ ^]^ in addition to exciting physical properties such as a gigantic Rashba splitting, and strong exciton–polariton interactions.^[^ ^10^, ^11^, ^12^ ^]^

Assessing the nature of the electronic species in close relation to the chemical structure of 2DP is of utmost interest with profound implications for excitonic devices such as quantum‐well modulators, light‐emitting devices, lasers, etc. Initial works from Tanaka et al.^[^ ^13^, ^14^ ^]^ identified and classified the exciton populations in standard 2D HP using electroabsorption (EA) spectroscopy as: i) Wannier‐type excitons (1s exciton) with large binding energies (>350 meV) causing a Stark effect manifested as a first derivative of the absorption line shape at the exciton resonance, a common characteristic for 2DP;^[^ ^15^ ^]^ ii) weakly bound excitons (i.e., with binding energies of the order of tens of meV, for higher Rydberg components) that undergo ionization under an applied electric field, resulting in a second‐derivative EA signal line shape induced by the exciton broadening.^[^ ^14^ ^]^ This is consistent with the behavior of quasi‐2D systems consisting of multiple *n* layers, where the quantum confinement is reduced and consequently, the exciton binding energy is lowered.^[^ ^15^ ^]^ In this case, a second derivative in the EA is indeed observed.^[^ ^13^, ^16^ ^]^ More recently, in such mixed systems, augmented separation of the electron and hole wave functions caused by the orientational dynamics of the small cation has been observed as an anomalous quantum‐confined Stark effect (QCSE) (blueshifting).^[^ ^12^ ^]^ In this work, we provide a comprehensive analysis that reconciles these conflicting views by focusing on a newly synthesized fluorine‐containing 2DP of high interest for stable and efficient perovskite devices.^[^ ^17^, ^18^ ^]^ Field‐induced changes in the linear optical absorption reveal a combination of first‐ and second‐derivative contributions at the exciton resonance. While the first‐derivative signal (redshifting) results from QCSE, the second‐derivative contribution is ascribed to charge pairs with spatially separated electrons and holes, as observed in other layered QW structures.^[^ ^19^, ^20^ ^]^ Notably, upon the generation of charges by the impulsive light excitation, a Stark effect is also observed due to excitons screening, lasting for microseconds. Ab initio computational modeling suggests that the combined effects of structural and electrostatic disorder induced by the fluorinated cations stabilize long‐lived weakly correlated charge pairs in these 2DP.

## 2 Results and Discussions

### 2.1 Optical Characterization

**Figure 1:**
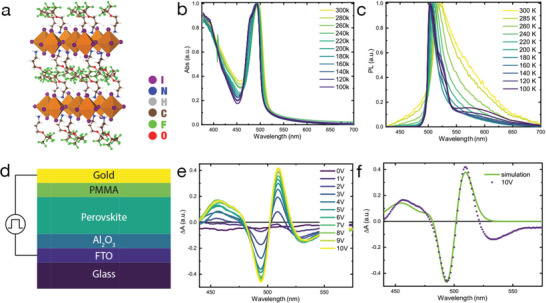
a) Cartoon showing the structure of (Fluo)_2_PbI_4_; b,c) Linear absorption and photoluminescence spectra (upon exciting at 420 nm) of (Fluo)_2_PbI_4_ versus temperature as indicated in the legend. d) Sketch of the device employed in electroabsorption (EA) experiments done at room temperature. e) EA spectra of the (Fluo)_2_PbI_4_ device at different biases. f) EA spectra at 10 V with a simulation composed of a linear combination of derivatives of the absorption bands.

**Figure** **1**a shows the structure of the fluorous‐2DP ((Fluo)_2_PbI_4_ hereafter), where a bulky fluorous cation (Fluo = (CF_3_)_3_CO(CH_2_)_3_NH_3_^+^) intercalates in between single inorganic layers adopting a pure 2DP (Fluo)_2_PbI_4_ structure.^[17]^ This is an attractive arrangement because of the resulting strong hydrophobic character, thereby creating a barrier layer against water penetration and allowing efficient and stable 2D/3D perovskite solar cells.^[^ ^21^, ^22^, ^23^ ^]^ In addition, this class of materials is also of high interest for their exceptional ease of tuning the exciton binding energy by fashioning the size and the length of the fluorous cation.^[^ ^18^ ^]^

The absorption profile of (Fluo)_2_PbI_4_ films (Figure 1b) shows a strong excitonic peak at the band edge at 495 nm (2.5 eV), which sharpens going down in temperature. As discussed by Neutzner et al., a clear second peak is observed at low temperature, in this case at 498 nm, separated by a 51 meV energy gap to the main exciton band.^[^ ^24^ ^]^ This is the fingerprint of a hidden exciton characterized by a different coupling to the lattice.^[^ ^25^ ^]^ From Tauc plot analysis (Figure S1, Supporting Information), the electronic band‐to‐band transition is estimated around 427 nm (2.9 eV), which indicates an exciton binding energy of around 400 meV. Similarly, the emission shows a single peak, excitonic in nature, which gets narrower going down in temperature (see PL spectra in Figure 1c). In addition, a broader in‐gap band can also be identified at low temperature, which we tentatively assign to the formation of local defects acting as color centers.^[^ ^26^ ^]^ We also note the absence of a shift (or a discontinuity) of the band edge with temperature, indicative of no phase transition in the temperature range investigated. This contrasts with standard 2DP (see, for instance, butyl ammonium based 2DP, used as a reference, reported in Figure S2, Supporting Information). The modeled absorption of (Fluo)_2_PbI_4_, reconstructed following the model by Kattoor et al., i.e., by using a linear combination of Gaussian curves to simulate the excitonic transitions as well as the continuum, is shown in Figure S1 (Supporting Information).^[^ ^27^ ^]^

### 2.2 Electroabsorption

To get a deeper understanding of the nature of the electronic excitations, we measured the EA spectrum (Figure 1e) of a (Fluo)_2_PbI_4_ perovskite film in a device configuration, as shown in the cartoon at Figure 1d done at room temperature. Figure 1e displays the EA signal at different bias applied perpendicular to the film substrate. In light of the strong crystalline orientation of the thin film lying parallel to the substrate, this should mainly correspond to the direction perpendicular to the inorganic PbI_4_ sheets within the film, thus along the axis of confinement (see XRD in Figure S3, Supporting Information). The EA spectrum exhibits a modulation consisting of a negative peak at 495 nm and a positive peak at 508 nm, respectively, along with two sidebands: one positive at 455 nm and one negative at 540 nm. For quantum‐confined semiconductors, the EA spectrum can be modeled in the framework of Stark's theory.^[^ ^28^ ^]^ More in details, the EA can be decomposed into a linear combination of first‐ and second‐derivative contributions to the linear absorption spectrum, whose relative amplitudes provide insight into the type of carriers subjected to the perturbation. Interestingly, in this case, the modulation can be well simulated with a 2:1 ratio of first and second derivatives of the excitonic peak (see Figure 1f). Note that the fit is poor in the low‐energy part of the spectrum. In this region below the gap, a broad, featureless negative signal appears which is related to shallow trap states in 2D perovskite, in agreement with what is already discussed in ref. [11], which the derivative fit does not consider. The first‐derivative contribution is assigned to QCSE (redshifting), while the second derivative indicates the formation of screened electron–hole pairs.^[^ ^29^ ^]^ In addition, its shape calls for loosely bound electron–hole pairs with predominant charge‐transfer character (CTC). This suggests that electrons and holes can separate in the [PbI_6_]^4−^ inorganic wells, in contrast to common classification on 2DP.^[^ ^29^ ^]^

### 2.3 Nanosecond Transient Absorption

Time‐resolved transient absorption measurements in the nanosecond domain have been performed to elucidate the dynamic response of the system upon impulsive excitation, which generates a high carrier density of around 10^18^ cm^−3^.

**Figure 2:**
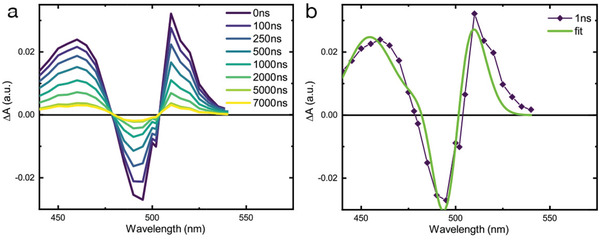
a) ns transient absorption (nsTA) spectra at selected delays (as indicated in the legend) for the (Fluo)_2_PbI_4_ thin film upon excitation at 420 nm, carrier density 10^18^ cm^−3^ b) nsTA signal at 1 ns probe delay with the simulated EA spectra (as in Figure 1f).

We show in **Figure** **2**a the ns transient absorption (nsTA) spectra of the (Fluo)_2_PbI_4_ thin film at selected pump‐probe delays. The spectra exhibit an oscillatory feature with two positive peaks at 460 and 510 nm as well as a negative peak at 495 nm. It is apparent that the spectral evolution of the nsTA resembles a combination of derivative‐like features with two zero‐crossing points that do not move in time. They all exhibit the same decay, showing a faster component with a time constant of τ_1_ = 930 ns and a second τ_2_ extending beyond our temporal window. To further analyze the spectral behavior and retrieve the associated time constant, we fitted the whole spectral evolution using a global analysis (GA) procedure (Figure S5, Supporting Information). The three bands have the same dynamics, suggesting that one process is responsible for the whole spectral evolution. Figure 2b shows a cut of the nsTA signal at 1 ns delay superimposed with the linear fit of the EA data (Figure 1f). Notably, for nsTA, no field is externally applied, thus the perturbation related to a long‐lived photoinduced Stark effect in this case only arises upon illumination and consequent creation of long‐lived charge pairs. It is worth pointing out that this observation differs from previous reports on alkylammonium‐based 2DPs (e.g., (C_6_PbI_4_) containing hexylammonium cations), where only a first‐derivative contribution in the EA spectra was reported and associated with strongly bound excitons.^[^ ^5^ ^]^ In our case, despite the very similar exciton binding energy (400 meV in (Fluo)_2_PbI_4_ vs 360 meV in C_6_PbI_4_), an additional contribution from long‐lived, loosely bound, charge pairs is observed.

### 2.4 Computational Investigation

**Figure 3:**
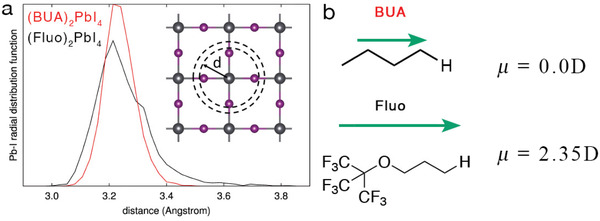
Results of frozen glass simulation. a) Lead‐iodide radial distribution function computed for (Fluo)_2_PbI_4_ and (BUA)_2_PbI_4_. b) Dipole moment for BUA and Fluo cations, with the NH_3_^+^ group substituted by a hydrogen atom to preserve the charge neutrality.

Since the physical origin of the screening of the correlated electron–hole pairs is inherent to the detailed chemical composition of the systems, we have performed periodic density functional theory (DFT) calculations using the planewave/pseudopotential formalism, as implemented in the Quantum Espresso package (see Computational Methods for a detailed description of the approach).^[^ ^30^ ^]^ We performed preliminary calculations on these systems considering the crystalline models reported in literature.^[^ ^17^, ^31^ ^]^ The corresponding partial density of states (pDOS) of (Fluo)_2_PbI_4_ is reported in Figure S6 (Supporting Information) and shows somewhat larger bandgap compared to reference (BUA)_2_PbI_4_, a fact that has been directly related to larger rotation of the PbI_6_ octahedra in the former system, as discussed in ref. [18]. However, the qualitative trend in the pDOS is similar, consistent with the fact that the models from the XRD experimental structures share similar Pb—I bond length and structure. Most notably, we notice that both the Fluo and BUA present a type I electronic alignment between the inorganic and organic frame, hence with the frontier levels of the former embedded in the frontier levels of the latter component. So, for both systems we can exclude direct perturbation of the PbI_4_ electronic structure due to the cation, as manifested by the absence of intragap trap states. Rather, the impact of the organic cations is twofold: i) a (indirect) structural effect. As well‐known from literature, the organic cation can impact on the electronic structure of the inorganic semiconducting component through distortion of the octahedral lattice.^[^ ^32^, ^33^, ^34^ ^]^ This is expected to be particularly important here as a result of the bulky character of the C—(CF_3_)_3_ fluorous spacers (see Figure 1a). ii) A (direct) electrostatic effect. The decoration of the chains with strongly electronegative fluorine atoms pointing away from the inorganic lattice is expected to yield a sizeable electrostatic potential affecting the energy landscape explored by holes and electrons residing within the PbI_4_ layers. To address the possible role of an indirect structural effect, we performed frozen‐glass simulations as follows. We first carried out ab initio Born–Oppenheimer molecular dynamics (BOMD) simulations at high temperature (600 K, see the Experimental Section in the Supporting Information for details), so as to widely explore the ground‐state potential energy surface.^[^ ^39^, ^40^, ^41^, ^42^ ^]^ Then, we randomly picked up 40 structures from the MD trajectory, which were fully minimized (at 0 K). Using such a numerical thermal annealing protocol, we do not allow the system to reach the absolute minimum energy configuration, but rather freeze the system into a set of local minima on the ground‐state potential energy surface. In **Figure** **3**a, we compare the lead‐iodine radial distribution function for the so‐prepared frozen glasses of the fluorous‐based perovskite, as compared to butylammonium (named BUA after‐on) taken as reference material. BUA shows the usual distribution of bond lengths among the inorganic atoms,^[^ ^35^, ^36^ ^]^ with the first peak, associated to the lead‐to‐iodine distance, peaking at the equilibrium distance of ≈3.2 Å (full width half maximum, FWHM is 0.12 Å). Most interestingly, in the case of the fluorous cations, the Pb—I distance, while being centered at the same value, shows a significantly broader distribution (FWHM of 0.18 Å). In particular, configurations with lead‐to‐iodine distances in excess of 3.75 Å, hence 0.55 Å longer than the typical Pb—I distances are now accessible. Thus, compared to BUA, the bulky fluorous cations distort more severely the PbI_4_ inorganic structure. It is also worth pointing out that the single‐crystal structure revealed a triclinic symmetry in contrast to the monoclinic structure for the most common 2D perovskite, already indicating a more distorted structure.^[^ ^18^ ^]^ Most noteworthy, previous literature in the field already pointed out the importance of structural dynamics and local distortions in affecting the electronic features of hybrid halide perovskites, as related, for instance, to the variation of the optical properties with temperature,^[^ ^36^ ^]^ as first step toward the formation of point defects,^[^ ^37^ ^]^ to explain less effective electron–phonon scattering events.^[^ ^38^ ^]^

We now turn to the collective electrostatic effects of the cations on the energy of the frontier crystalline orbitals. In 2D slabs, an extended monolayer of oriented dipoles produces a jump (Δ*E*) in electrostatic potential with respect to vacuum, which can be cast in terms of the component of the dipole moment orthogonal to the slab surface (μ_z_) per unit area (A) via the Helmholtz relation

(1) $$\Delta E=\frac{-\mu_{\text{z}}}{\varepsilon_{0}A}$$

(where ε_0_ is the vacuum dielectric constant).

**Figure 4:**
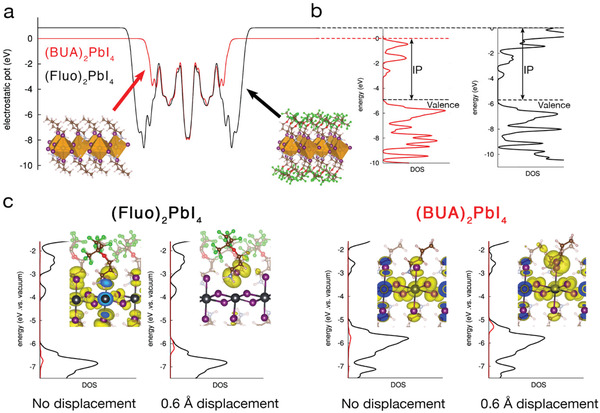
a) Electrostatic potential along the thickness of (BUA)_2_PbI_4_ and (Fluo)_2_PbI_4_ slabs; b) Partial density of electronic states (pDOS) for the two materials referred to the electrostatic potential in (a). The ionization potential (IP) is indicated. c) Total density of state (black curve) and density of state (red line) of one iodine which has been pulled out by 0.6 Å (vide infra) with respect to the central PbI_4_ plane. The spatial localization of the valence band edge orbital is reported in the inset.

As shown in Figure 3b and in agreement with our expectations, the fluorinated side group of the fluorous organic cation (Fluo, see Figure 3b) features a larger dipole than the corresponding hydrocarbon chain of BUA. Here, the calculations are performed by substituting NH_3_^+^ with H, as dipoles of charged molecules are ill‐defined (notably, similar results are obtained considering CH_3_ or NH_2_, terminal groups, see the Supporting Information). This is corroborated by the DFT electronic structure calculations reported in **Figure** **4**a, showing an upshift of the electrostatic potential in the vacuum region by ≈1 eV when going from (BUA)_2_PbI_4_ to (Fluo)_2_PbI_4_ (in line with the difference in dipole moments in Figure 3 and with Equation (1)). Correspondingly, the valence and conduction band edges of (Fluo)_2_PbI_4_ get stabilized by the same Δ*E* value, as shown in Figure 4b, with, namely, larger ionization potential (IP) for (Fluo)_2_PbI_4_, compared to the reference (BUA)_2_PbI_4_.

The combination of the electrostatic and structural distortion effects discussed before provides a simple rationale for the formation of charge‐separated states in (Fluo)_2_PbI_4_. Inspired by the results in Figure 3, namely, that the (Fluo)_2_PbI_4_ can sustain significant distortions of the Pb—I lattice, we propose that iodine atoms are more labile in the fluorinated compounds and, to evaluate the effects of such structural distortions on the electronic structure of the material, we performed “pull‐out” numerical experiments, where one iodine atom is increasingly dragged away from the inorganic lattice by the closest organic cation. Considering a displacement of 0.6 Å, which is on par with the range of values predicted by MD simulations in Figure 3a, for the apical‐iodine Fluo‐cation, we find that gap states located around the labile iodine ions form just above the valence band edge in the case of FluoPbI_4_ (see Figure 4c). We stress that it is the combined effect of the increased structural distortion prompted by the bulky side groups together with the overall downshifts of the delocalized band states driven by fluorine‐induced electrostatic effects that expels these shallow trap states from the band edge into gap. As a matter of fact, such localized trap states do not show up in similar simulations performed for (BUA)_2_PbI_4_, at least, considering 0.6 Å displacement. In Figures S7 and S8 (Supporting Information), we report both partial density of states and shape of the highest occupied orbital for (BUA)_2_PbI_4_ and (Fluo)_2_PbI_4_ materials, with increasing pull‐out displacements ranging from 0.15 to 0.6 Å. For the case of (Fluo)_2_PbI_4_, the appearance of shallow trap states, fully localized on the apical iodine interested in the pull‐out is already evident at 0.45 Å, while for the (BUA)_2_PbI_4_, these states still lie below the delocalized valence band maximum even for displacement 0.6 Å. Thus, following Figure 3a, we propose that the frozen glass arrangement of the side chains in the fluorous 2D perovskites can stabilize a broad range of configurations that differ by the relative displacement of some axial iodines with respect to the equatorial lead‐iodine layer. Such configurations would support electron–hole pair excitations with spatially separated wave functions, explaining their ionic (CT) character as revealed by EA as well as their long lifetimes measured by nsTA. We expect the spatial distribution of axial iodines to extend continuously from their crystalline equilibrium position up to full separation with respect to the inner layer, with the corresponding formation of electronic excitations going from confined excitonic states for small displacements to poorly overlapping electron–hole pairs in the limiting case of halide vacancies/interstitial Frenkel defects.^[^ ^26^ ^]^ This seems to be corroborated by the presence of the broad ≈600 nm emission in Figure 1c. The combination of the electrostatic and structural distortion effects discussed before provides a simple rationale for the formation of charge‐separated states in (Fluo)_2_PbI_4_. Indeed, larger structural flexibility of the fluorinated layered perovskite goes together with stronger electrostatic effect imposed by the larger molecular dipoles, in the normal direction to the inorganic PbI_4_ layer, as shown in Figure 4a,b. In this frame, we propose that structural distortions of the Pb—I lattice along the direction normal to the inorganic plane, hence in the direction of the strong variations of the electrostatic landscape in Figure 4a, strongly impacts on the formation of free carriers. Before concluding, we briefly discuss the role of oxygen present in the Fluo cation, in driving the formation of the trap states highlighted in Figure 4c. Indeed, we inherently attributed the electrostatic effect dictated Fluo cation, as quantified by the larger ionization potential computed for this system, only to the terminal fluorine atoms, but oxygen can also contribute to such an effect, in light of its larger electronegativity compared to carbon. To verify this, we performed a similar pull‐out computational procedure to a hypothetical new cation, having the same backbone of Fluo but with all the fluorine atoms substituted by hydrogens (we name this cation Fluo‐H). From the pDOS in Figure S9 (Supporting Information) it is evident that the removal of fluorines in the Fluo‐H cation results in ≈1 eV upshift of the ionization potential of the perovskite, compared to the original Fluo. As a result, the new fluorine‐free cation has ionization potential closer to the one of BUA. Consistently, inspection of the highest occupied orbital for Fluo‐H reveals that this corresponds to the delocalized valence band edge and no shallow trap states form for distortions up to 0.6 Å. In other words, the removal of fluorines results in a less effective electrostatic effect on the electronic structure of the PbI_4_ lattice significantly reducing the possibility of formation of shallow trap states, similar to what was found in the reference BUA material. Hence, in the perspective of cation engineering, the oxygen on the backbone of the organic molecule does not seem to be related to the optical response evinced from the EA measurements and on the long lifetime related to the Fluo cation.

## 3 Conclusion

In conclusion, our work provides compelling evidence for the existence of long‐lived weakly bound charge pairs in layered n = 1 (Fluo)_2_PbI_4_ 2DP as evident from the anomalous second derivative shape of the field (and light) induced Stark signal. Furthermore, the optical response of this material to an impulsive optical perturbation develops over hundreds of nanosecond timescale. This result is surprising in view of the large exciton binding energy measured in these 2D materials and highlights the role of the chemical composition of the organic component. Specifically, our atomistic calculations show that the presence of bulky C(CF_3_)_3_ groups in the fluorinated cation results in frozen‐glass like structures, where the apical iodines can explore a broad range of distances from the PbI‐inorganic layer, compared to standard alkylated materials. In addition, the fluorous side groups result in a sizeable electrostatically driven energy shift of the crystalline orbitals. It is the combined structural and electrostatic effects that promote the formation of spatially confined hole states at the valence band edge and stabilize loosely interacting electron–hole pairs with reduced recombination and longer lifetimes. While this feature is to our knowledge unique to Fluo‐based perovskite, our study broadens the standard classification of electronic species in 2DP and opens up a way for the design of new 2DP materials. Indeed, smart design of new organic cations, characterized by bulkier/more disordered structure and/or larger molecular dipole can potentially drive the system to larger fraction of long‐lived charge carriers, which are of interest for perovskite‐based 2D/3D photovoltaic devices, as well as for lasers and delayed emitters. Future time‐resolved, power‐dependent, and/or field‐dependent studies can further help to identify the nature of the excited species in 2D layered perovskites family and beyond.

## 4 Experimental Section

##### Sample Preparation

To fabricate (Fluo)_2_PbI_4_ thin films, the precursor solution was prepared by mixing a 2:1 molar ratio of the corresponding ammonium salt ((CF_3_)_3_CO(CH_2_)_3_NH_3_I), synthesized as previously reported,^[^ ^17^ ^]^ and PbI_2_ in DMSO. The one‐step deposition method using chlorobenzene as antisolvent was used. The thin film was annealed at 100 °C for 15 min. This results in a 300 nm thick film.

##### Characterization

Steady‐state absorption spectra were acquired with a Perkins Elmer Lambda 950s UV–vis spectrophotometer using an integrating sphere to account for optical losses outside of the active layer.

Steady‐state and time‐resolved photoluminescence measurements were carried out on a Horiba a Fluorolog‐3, with a PMT as detector. The excitation source for the TCSPC is a Horiba nanoLED‐370 with an excitation wavelength of 369 nm, a pulse duration of 1.3 ns, and a repetition rate of 100 MHz.

Low‐temperature PL and absorption were performed in their respective instruments fitted with a OptistatND from Oxford Instruments.

##### X‐Ray Diffraction

XRD patterns were recorded by X‐ray diffractometer (Bruker D8) with Cu Kα radiation.

##### Nanosecond Transient Absorption

Nanosecond transient absorption measurements were carried out with a LP980 laser flash spectrometer (Edinburgh Instruments). It is based on a standard transient absorption setup where the sample is excited by a ns laser pulse and the time evolution of the differential absorption changes induced by the pump is monitored by a CW light source probe. The pump pulses were provided by a nanosecond tunable Ekspla NT340 laser (5 Hz repetition rate). The excitation wavelength was set at 420 nm and the excitation density was tuned from 4 to 40 µJ cm^−2^ but negligible effects on the dynamics were observed on these time scales. The probe light was provided by a pulsed Xenon arc lamp. The sample was kept at a 45° angle to the excitation beam. The beams were focused onto the sample ensuring spatial overlap. The transmitted probe was spectrally filtered by a monochromator and detected. The detection systems is based on a set of photomultipliers (with both vis and near‐IR detection window) enabling one to collect the single‐wavelength kinetic with higher sensitivity. The signal was finally recorded by a TDS 3032C digital signal analyzer. From the transmission change following photoexcitation the variation in the absorption is thus derived as

(2) $$\Delta \text{OD}\left(\tau ,\lambda \right)=\log\left(\frac{I_{\text{probe}}}{I_{\text{t}}\left(\tau ,\lambda \right)}\right)$$

where *I*_probe_ is the transmitted probe with excitation off and *I*_t_ is the transmitted probe after laser excitation. The system has a sensitivity of ≈5 × 10^−4^ V and a temporal resolution of ≈5 ns.

##### Electroabsorption

Electroabsorption spectroscopy measurements consist of detecting a probing light beam after its interaction with a sample subjected to an externally applied electric field done at room temperature. In the present case, EAS measurements were performed on a common Ti:Sapphire amplified femtosecond laser system by Clark‐MXR (CPA‐2001), yielding 780 nm pulses at a repetition rate of 1 kHz. The probe beam was obtained by passing the 780 nm laser output through a sapphire plate yielding a white light continuum detected over the 400–850 nm region. After being transmitted through the sample (transmittance mode, semitransparent gold electrode) or being reflected off of its gold electrode (reflectance mode), the probe beam was dispersed in a grating spectrograph (SpectraPro 2500i, Princeton Instruments or SR163, Andor Technology) and finally detected shot by shot at a 1 kHz rate with a 512 × 58 pixel back‐thinned CCD detector (S07030‐ 0906, Hamamatsu). Part of the probe beam was split before the sample into a reference beam reaching a second detector, which allowed for corrections for shot‐to‐shot fluctuations. The externally applied voltage was controlled by a function generator (AFG 2021, Tektronix), yielding square voltage pulses (100 µs pulse duration). The voltage pulses were modulated at 500 Hz, allowing to get the desired differential signal DA = *A*_field_ − *A*_nofield_. Multiple samples were measured under the same conditions, yielding consistent results.

##### Computation

Both the frozen glass annealing simulation (consisting in Born–Oppenheimer molecular dynamic simulations and subsequent structural relaxations at 0 K temperature) and the electronic structure calculations on slabs models were performed using periodic DFT calculations, within the planewave/pseudopotential formalism, as implemented in the Quantum Espresso Suite.^[^ ^30^ ^]^ All calculations were performed using a cutoff of 25 and 200 Ry, for the expansion of the wavefunction and of the electron density, respectively, along with ultrasoft pseudopotentials^[^ ^43^ ^]^ and PBE potential^[^ ^44^ ^]^ for the description of the exchange‐correlation energy. DFT‐D2 scheme was used^[^ ^45^ ^]^ to improve the description of the van der Waals interactions between the organic molecular spacers.

BOMD simulations and structural relaxations were performed on 2 × 2 × 1 supercells of the structural models of (BUA)_2_PbI_4_ and (Fluo)_2_PbI_4_ available from refs. [31] and [18], respectively. BOMD simulations were performed using a time step of 20 a.u. (≈1 fs) at 600 K temperature for both (BUA)_2_PbI_4_ and (Fluo)_2_PbI_4_, so to widely explore the potential energy surface of the materials. To further speed up the sampling of the potential energy surface, the effective masses were decreased to 10 a.u., for the inorganic atoms, 5 a.u. for C, O, N, F and 2 a.u. for H. Notice that this is a common protocol in meta‐dynamic simulations and does not affect the quality of the potential energy surface, since it does not influence the interatomic forces.^[^ ^41^ ^]^ In light of the large size of the structural models employed (624 and 472 atoms, respectively, for (BUA)_2_PbI_4_ and (Fluo)_2_PbI_4_), the electronic structure was evaluated only at the Γ point of the reciprocal space.

Slab calculations were carried out isolating one slab from the crystalline cells of (BUA)_2_PbI_4_ and (Fluo)_2_PbI_4_ providing 15 Å between the slab in the periodic cell and its periodic replica. For these calculations, the dipole correction was added, in light of the structural asymmetry introduced during the pull‐out computational experiment. For these calculations, the reciprocal lattice was sampled using an automatic 4 × 4 × 1 grid,^[^ ^46^ ^]^ with the less dense sampling associated to the direction of the plane stacking.

## Conflict of Interest

The authors declare no conflict of interest.

## Supporting information

Supporting Information
