# Supplementary material for: Spatial Charge Separation as the Origin of Anomalous Stark Effect in Fluorous 2D Hybrid Perovskites
Source: Adv Funct Mater. 2020 May 28;30(28):2000228. doi: 10.1002/adfm.202000228 (PMC7357595; doi:10.1002/adfm.202000228)
Supplement: Supplementary file 1 — Supporting Information [file ADFM-30-2000228-s001.pdf]

Copyright WILEY-VCH Verlag GmbH & Co. KGaA, 69469 Weinheim, Germany, 2020.

ADVANCED FUNCTIONAL MATERIALS

Supporting Information

for *Adv. Funct. Mater.,* DOI: 10.1002/adfm.202000228

Spatial Charge Separation as the Origin of Anomalous Stark Effect in Fluorous 2D Hybrid Perovskites

Valentin I. E. Queloz, Marine E. F. Bouduban, Ines García-Benito, Alexander Fedorovskiy, Simonetta Orlandi, Marco Cavazzini, Gianluca Pozzi, Harsh Trivedi, Doru C. Lupascu, David Beljonne, Jaques-E Moser, Mohammad Khaja Nazeeruddin, Claudio Quarti, and Giulia Grancini**

Supporting Information

Spatial Charge Separation as the Origin of Anomalous Stark Effect in Fluorous Two-Dimensional Hybrid Perovskites

Valentin I. E. Queloz[1], Marine E. F. Bouduban[2], Inés García-Benito[1], Alexander Fedorovskiy[1], Simonetta Orlandi[3], Marco Cavazzini[3], Gianluca Pozzi[3], Harsh Trivedi[4], Doru Lupascu[4], David Beljonne[5,] Jacques-E. Moser[2], Mohammad Khaja Nazeeruddin[1], Claudio Quarti,[5,6*] Giulia Grancini[1,7*]

[1] Group for Molecular Engineering of Functional Materials, Institute of Chemical Sciences and Engineering, Ecole Polytéchnique Fédérale de Lausanne, Sion CH-1951, Switzerland.

[2] Photochemical Dynamics Group, Institute of Chemical Sciences & Engineering and Lausanne Centre for Ultrafast Science (LACUS), École Polytéchnique Fédérale de Lausanne, CH-1015. Lausanne, Switzerland

[3] Consiglio Nazionale delle Ricerche, Istituto di Scienze e Tecnologie Chimiche "Giulio Natta" (CNR-SCITEC), Via Golgi 19, I-20133 Milano, Italy

[4] Institut für Materialwissenschaft, Fakultät für Ingenieurwissenschaften, Universität Duisburg-Essen, Raum V15 S05 D06 Universitätsstraße 15, 45141 Essen, Germany

[5] Laboratory for Chemistry of Novel Materials, University of Mons, Place du Parc 20, B-7000 Mons, Belgium

[6] University of Rennes, ENSCR, INSA Rennes, CNRS, ISCR (Institut des Sciences Chimiques de Rennes) UMR 6226, F-35000 Rennes, France

[7] Department of Chemistry, University of Pavia, Via Taramelli 14, 27100 Pavia, Italy

*Correspondence to: claudio.quarti@umons.ac.be; giulia.grancini@unipv.it.

Keywords : Electroabsoprtion, transient absorption, 2D perovskite, stark effect

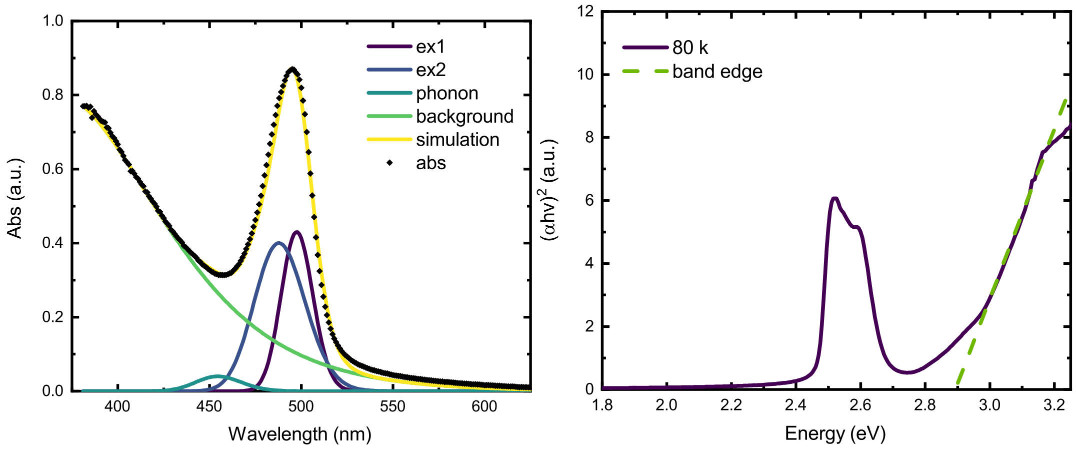

Figure SI1.Fit of the absorption of $(Fluo)_2PbI_4$ at room temperature with 5 gaussians (left). Tauc plot of $(Fluo)_2PbI_4$ at 80K(right).

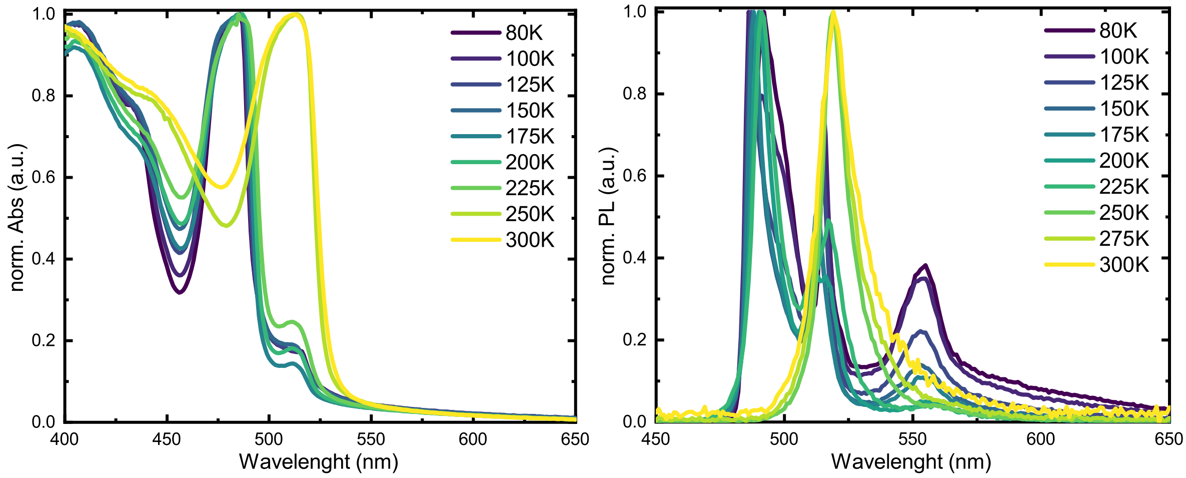

Figure SI2. Normalized absorption vs. temperature of $(BUA)_2PbI_4$ (left).PL vs temperature of $(BUA)_2PbI_4$ (right).

Both in the absorption and PL spectra we can clearly see some discontinuities between two temperatures. This is the signature of phase transitions.

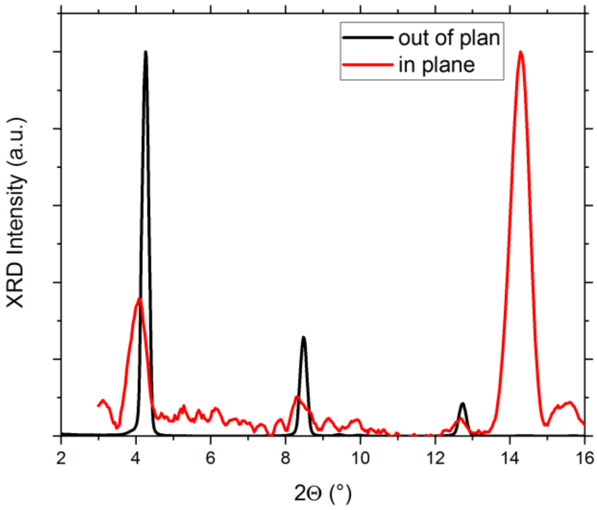

Figure SI3. In plane and out of plane X-ray Diffraxtion of the thin film of $Fluo_2PbI_4$ perovskite.

XRD of $(Fluo)_2PbI_4$ thin film showing two shape peaks at 2 Θ = 4 and 8.5. These two peaks are characteristic of 2D layered perovskites. Moreover, the intensity of the out of plane peaks compared to the in plane ones suggests that the inorganic planes have a strong preferred orientation parallel to the substrate surface.

1.1. EA contribution to the signal

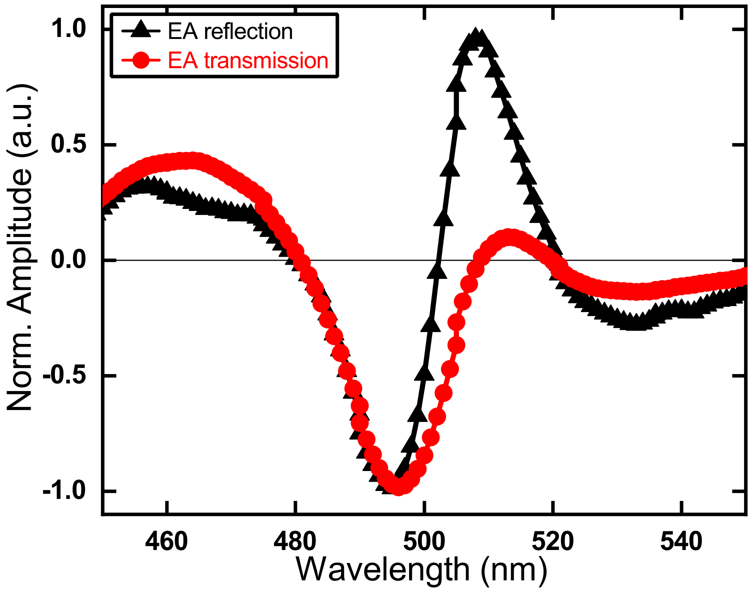

Figure SI4. EA in reflection and transmission mode at 10V.

From perturbation theory, one can define the EA signal as (perturbative expansion in the field):

$$\Delta A = -\frac{\partial A}{\partial \epsilon} m_{0k}\mathbf{E} - \frac{1}{2}\frac{\partial A}{\partial \epsilon} p_{0k}\mathbf{E}^2 + \frac{1}{2}\frac{\partial^2 A}{\partial \epsilon^2}(m_{0k}\mathbf{E})^2 + \ldots$$

(1)

where A denotes the absorbance, E describes the applied electric field and m_{0k} and p_{0k} respectively correspond to the permanent dipole moment and polarizability changes upon the transition of interest. Note that the first term (linear in the field) is expected to cancel out for isotropic samples.

EAS Measurements in Reflectance vs. Transmittance:
EAS measurement have been done in both transmission and reflection modes (see Figure SI4). The spectral difference comes from the gold electrode. In this case the reflection spectra can be considered as a double reflection. The transmission spectra amplitude around 515nm is attenuated, because the probe beam has to go through the gold which strongly absorbs close to this wavelength.

1.2. Global Analysis

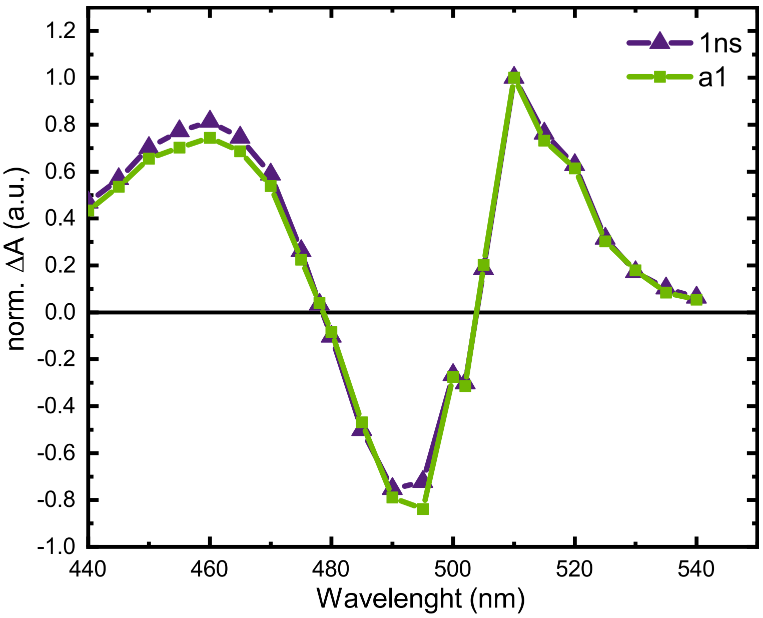

Figure SI5. Superposition of the TA spectra at 1ns delay with the amplitude of the exponential fit from the global analysis, both normalized.

$$f(x) = a1 * e^{x/\tau} + c$$

(eq 2)

To further analyze the spectral behavior and retrieve the associated time-constant, we fitted the whole spectral evolution using a Global Analysis (GA) procedure.
Representation of the data by the GA The spectral evolution over the whole frequency range can be fitted considering an exponential function with amplitudes represented in Figure SI5 and time constant t=932ns and a constant that acts as a very long time constant much longer that our time window (Eq. 2). These observations indicate that the three

features have the same dynamics, suggesting that one process is responsible for whole spectral evolution. Remarkably, it is the first time that such a long-lived signal has been reported for 2D HP.

1.3. Partial Density of States of Fluo2PbI4 and BUA2PbI4 perovskites

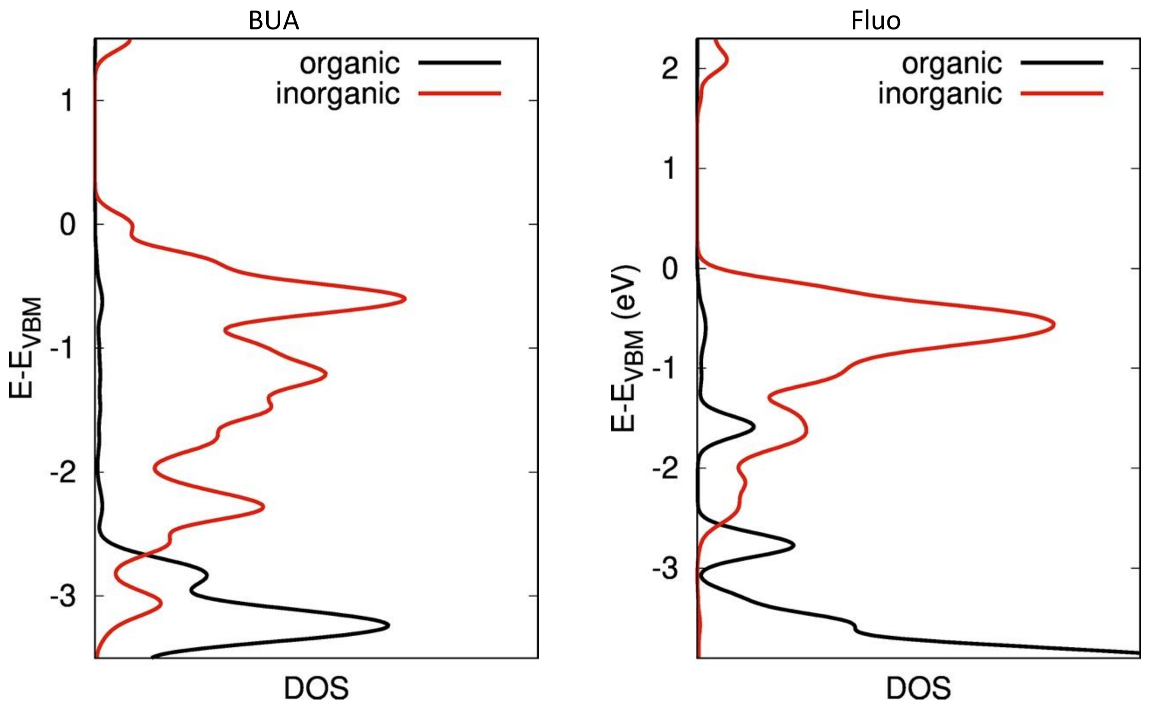

Fig. SI 6. Partial Density of States (pDOS) from DFT calculations performed on reference 2DP $(BUA)_2PbI_4$ and $(Fluo)_2PbI_4$ materials. The calculations are carried out on the models from XRD experimental measurement. The zero energy corresponds to the valence band edge. The present pDOS distinguishes between contributions from the inorganic lattice (red) and organic cation (black). The present calculations include Spin-Orbit Coupling (SOC).

1.4. Dipole computed for the BUA and Fluo molecules

Table SI1. Molecular dipole associated to the buthylammonium (BUA) and Fluorinated (Fluo) organic spacers. The NH_3^+ group has been substituted with H, CH_3 and NH_2 (see Figure 3b of the main text) to preserve the neutrality of the molecule (the dipole is ill-defined for non-neutral systems). For the sake of completeness, also the molecular dipole for molecules containing the group NH_3^+ is reported. Dipoles are computed using Gaussian16[44], adopting two exchange correlations functionals (PBE and B3LYP) along with 6-311G(d,p) triple zeta split basis set quality.

	H	CH_3	NH_2	NH_3^+
		PBE		
BUA	0.00	0.00	0.15	8.42
Fluo	2.35	2.25	2.78	20.26
		B3LYP		
BUA	0.00	0.00	0.26	8.87
Fluo	2.53	2.45	2.94	20.02

1.5. Pullout experiment with larger displacement for both Fluo2PbI4 and

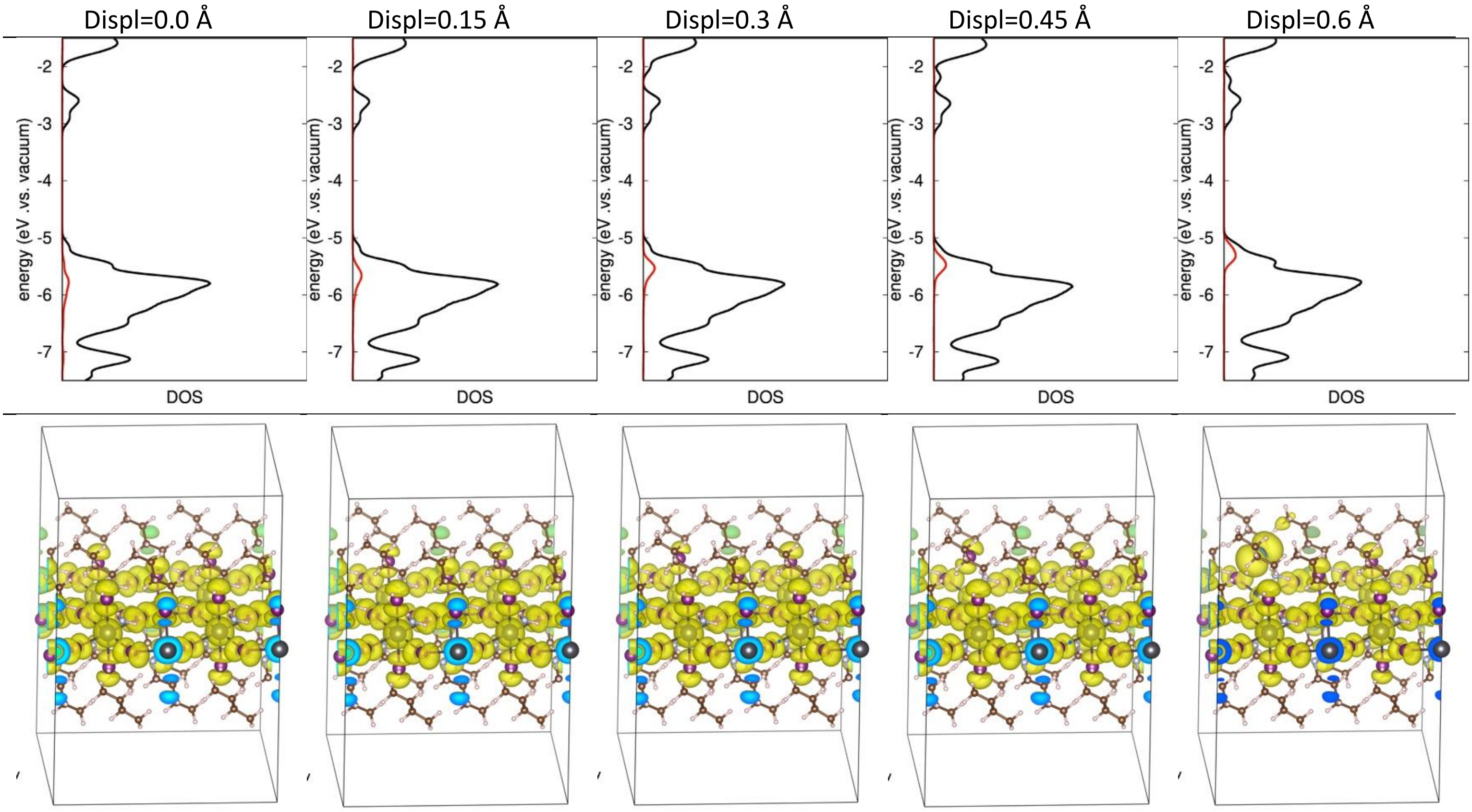

Figure SI7. Results of progressive pull-out of one apical iodine, along with one organic cation from the equilibrium position, for $(BUA)_2PbI_4$ 2D perovskite. Upper panel: total Density of States of (black line) and DOS associated with the apical Iodine (red line); bottom panel: crystalline orbital iso-density associated with the apical iodine involved in the pullout.

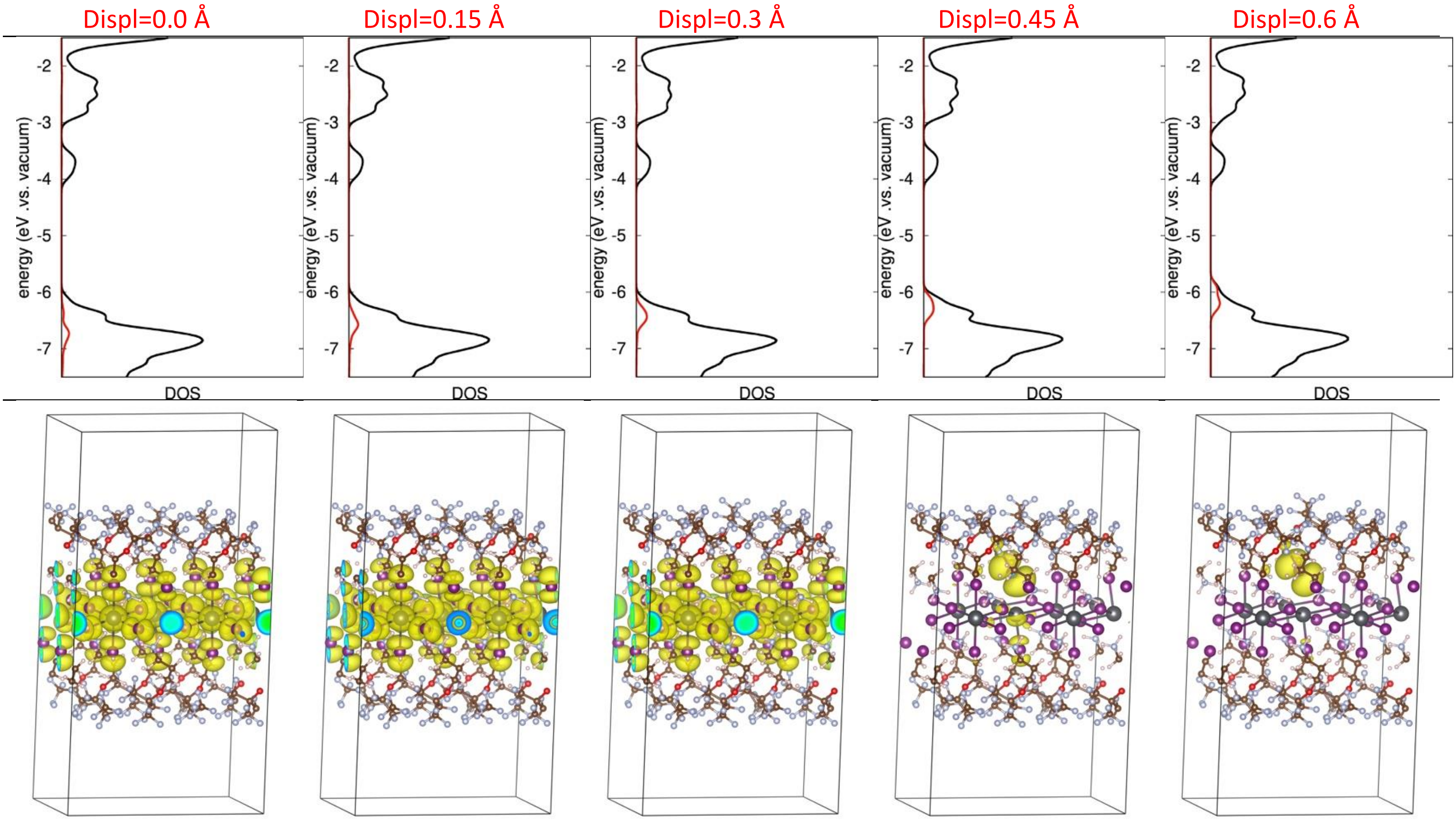

Figure SI8. Results of progressive pull-out of one apical iodine, along with one organic cation, from the equilibrium position, for $(Fluo)_2PbI_4$ 2D perovskite. Upper panel: total Density of States of (black line) and DOS associated to the apical Iodine (red line); bottom panel: crystalline orbital iso-density associated with the apical iodine involved in the pullout.

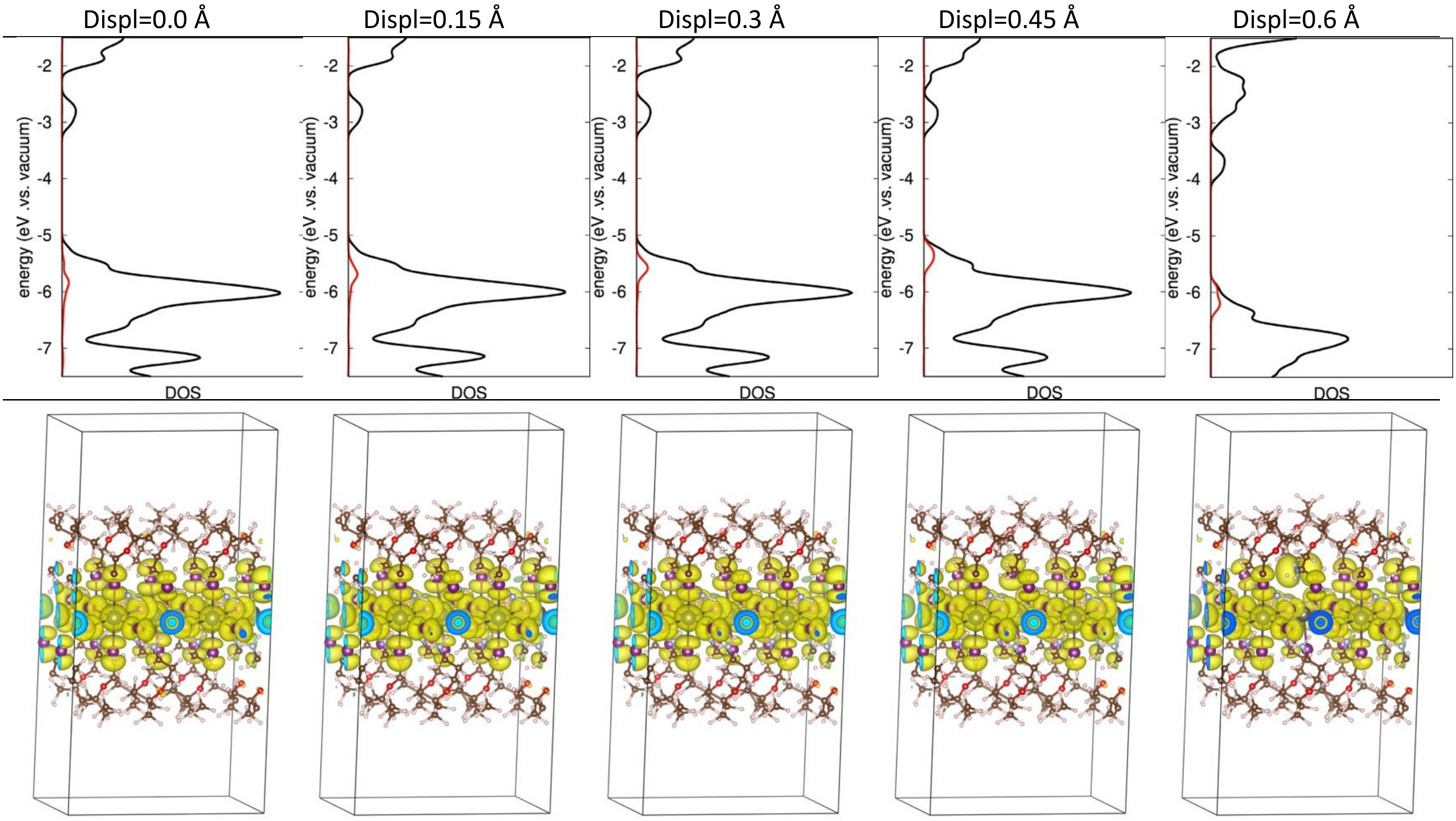

Figure SI9. Results of progressive pull-out of one apical iodine, along with one organic cation, from the equilibrium position, for $(Fluo\text{-}H)_2PbI_4$ 2D perovskite. The Fluo-H cation has the same structure as the Fluo but with fluorine atoms substituted by hydrogens. Upper panel: total Density of States of (black line) and DOS associated to the apical Iodine (red line); bottom panel: crystalline orbital iso-density associated with the apical iodine involved in the pullout.
